# Low Levels of Tumor Necrosis Factor-α will Prevent Periodontitis Exacerbation in Type 2 Diabetes Mellitus

**DOI:** 10.1055/s-0041-1739442

**Published:** 2022-01-11

**Authors:** Titiek Berniyanti, Gilang Rasuna Sabdho Wening, Retno Palupi, Dini Setyowati, Cindy Ramadhan Putri

**Affiliations:** 1 Department of Dental Public Health, Faculty of Dental Medicine, Universitas Airlangga, Surabaya, Indonesia; 2 Faculty of Dental Medicine, Universitas Airlangga, Surabaya, Indonesia

**Keywords:** healthy lifestyle, TNF-α, diabetes, periodontitis, behavior

## Abstract

**Objectives** Diabetes mellitus (DM) is a major risk factor for periodontitis. Susceptibility to periodontitis increases approximately three times in people with DM. There is a clear relationship between the degree of hyperglycemia and the severity of periodontitis. This study aimed to analyze the reduction of tumor necrosis factor-α (TNF-α) in diabetics who came for periodontitis examination to prevent exacerbations.

**Materials and Methods** This was an analytic observational study using a cross-sectional approach at health centers in Surabaya, Indonesia. Measurement of periodontal status used the community periodontal index of treatment needs by measuring bleeding at probing and pocket depth. TNF-α was measured using enzyme-linked immunosorbent assay, and behavior and lifestyle using a questionnaire.

**Statistical Analysis** The Kolmogorov–Smirnov test was performed to identify data normality ( *p* < 0.05). A nonparametric test was used to measure the degree of association between different characteristics and the incidence of periodontitis in type 2 DM patients with and without periodontitis. Spearman's test was done to examine the correlation between TNF-α level and severity of periodontitis in diabetics. The significant level was at *p* <0.05.

**Results** There was a correlation between age, predisposing factors, reinforcing factors, drug consumption, and TNF-α levels in patients with type 2 DM and the incidence of periodontitis.

**Conclusions** Poor glycemic control can induce oxidative stress on the gingiva, thereby aggravating damage to periodontal tissue. An important factor in preventing periodontitis for type 2 DM patients is controlling blood sugar levels through regular consumption of drugs and regular maintenance of oral cavity health. Knowledge is a predisposing factor that affects adherence of people with type 2 DM to consuming drugs regularly, which can be strengthened by family support. These will ultimately play a role in reducing TNF-α levels.

## Introduction

Diabetes mellitus (DM) is a metabolic disorder characterized by hyperglycemia due to abnormal insulin secretion. The common symptoms of DM are polyuria, polydipsia, polyphagia, and weight loss. 1 People with DM in the world in 2013 were 382 million and will continue to increase up to 592 million in 2035. 2 3 Basic Health Research in 2013 reported that the prevalence of DM in Indonesia increased from 1.1% in 2007 to 2.1% in 2013, and approximately 95% of them were type 2 DM, which tended to be caused by an unhealthy lifestyle. Type 2 DM can be prevented by controlling risk factors such as people's behavior and lifestyle. Periodontitis is the most common complication in DM patients with a prevalence rate of 75%. Based on the study, there were 118 people (93.7%) of 126 DM patients in 2008 who suffered from periodontitis. 4 It is an inflammatory process that occurs in the supporting tissues of the teeth in response to the accumulation of bacteria, or plaque on the teeth, and affects the adhesion of the connective tissue and supporting bone around the teeth. 5 Several studies have shown that the severity of periodontitis in DM patients is greater, especially in uncontrolled patients.

DM is classified into three types. Type 1 DM usually occurs in adolescents or children due to cell damage that is thought to be due to an autoimmune process. Type 2 DM in adults is due to decreased insulin receptor sensitivity resulting from worsening risk factors, such as overweight and lack of physical activity. Then the third type is gestational DM (GDM)—a diabetes that is diagnosed during pregnancy with signs of hormonal hyperglycemia. 3

Criteria for determining DM are as follows: sugar levels >126 mg/dL, random blood sugar >200 mg/dL, and HbA1c examination every 2 to 3 months with DM criteria >6.5–7%. 6 7 8 Type 2 DM is strongly influenced by risk factors. These factors can be changed through behavior and lifestyle modification. Behavior is a person's reaction to a stimulus. The behavior occurs through a stimulus process in the organism and then the organism responds. Behavior and lifestyle play an important role in controlling type 2 diabetes and periodontitis. Behavior modification is very important to control blood glucose levels and reducing the prevalence of DM, both in Indonesia and around the world. 9 A person's health behavior is influenced by three factors: predisposing factors, enabling factors, and reinforcing factors. 9 10 According to the World Health Organization, a healthy lifestyle is a lifestyle that can reduce the risk of disease or premature death from disease, which takes into account certain factors that affect health, including diet and exercise. 3 Immune response has a great role in the severity of inflammation in periodontitis. 11 Bacteria produce inflammatory mediators, such as prostaglandins or cytokines, including tumor necrosis factor-α (TNF-α) and interleukin-1. These mediators stimulate the production and activation of enzymes so that they can damage gingival connective tissue and produce osteoclasts that can absorb bone. 12

Poor blood sugar control and increased advanced glycation end-product (AGE) formation induce oxidative stress in the gingiva which will slow the breakdown of the periodontium tissue. Increased levels of inflammatory cells in the gingival crevicular fluid (GCF) also make the periodontium tissue more susceptible to infection and cause bone damage. Apart from damaging the leukocytes, another complication of DM is blood-vessel thickening which limits the flow of nutrients in the body. A slow blood flow will reduce the body's ability to heal infections so that periodontitis will get worse in people with diabetes. These are the factors that affect the periodontium condition in people with diabetes. 13 This study aimed to analyze the reduction of TNF-α in people with diabetes who came for periodontitis examination to prevent exacerbations.

## Materials and Methods

This was an observational analytic study with a cross-sectional approach, conducted after obtaining ethical permission from the Faculty of Dental Medicine Universitas Airlangga, Surabaya, Indonesia, with Licensing Certificate Number 133/HRECC.FODM/VII/2018. A total of 90 DM type 2 respondents with and without periodontitis were taken as samples. This study conformed to the STROBE guidelines with a checklist attached as a proof.

All respondents were elderly in Surabaya suffering from type 2 DM. Medical facilities in Surabaya were selected using cluster random sampling to obtain six health centers with 90 respondents. The confidence level was 90%. The inclusion criteria of respondents established to prevent bias were elderly people aged >45 years and suffering from type 2 diabetes. Respondents who were willing to take part in this study signed informed consent. All respondents were asked to fill out a questionnaire consisting of the respondent's behavior (predisposing factors, enabling factors, and reinforcing factors) and the respondent's lifestyle (diet, physical activity, maintenance of the oral cavity, and smoking habits). The predisposing factors included respondents' knowledge about type 2 DM and its impact on oral health; the enabling factors include the availability of costs and access to health services; while the reinforcing factors included family support for respondents in maintaining their health.

After filling out the questionnaire, the respondents were checked for periodontal status using the community periodontal index of treatment needs (CPITN), which included the amount of bleeding on probing and the depth of the pocket. In addition, the TNF-α level was measured by ELISA (enzyme-linked immunosorbent assay) to the respondent's saliva. As much as 5 mL of saliva was produced through stimulation by chewing a tampon. The collected saliva was examined for TNF-α levels using an ELISA kit to determine the correlation between TNF-α levels and the incidence of periodontitis in type 2 DM patients.

For statistical analysis, the Kolmogorov–Smirnov test was done to identify data normality with *p* <0.05. A nonparametric test was used to measure the degree of correlation between different characteristics and the incidence of periodontitis in type 2 DM patients with and without periodontitis. Spearman's test was done to examine the correlation of TNF-α level and severity of periodontitis in those diabetics with a significance level of *p* <0.05.

## Result

Age was found to have a significant difference with *p* = 0.042 ( *p* < 0.05), showing that the older the patient, the higher the risk of type 2 DM. Whereas, the variables of the duration of suffering and family history did not have a significant correlation with the incidence of periodontitis in patients with type 2 DM ( Table 1 ). The predisposing factors, which were represented by knowledge, had a significant correlation with periodontitis ( *p* -value = 0.000, *p* < 0.05), and so did the reinforcing factors ( *p* = 0.012, *p* < 0.05). The enabling factors did not have a significant correlation with the incidence of periodontitis in patients with type 2 DM ( Table 2 ). A good diet with fruit and vegetable consumption was found not to have a significant correlation, and so were the physical activity, management of oral cavity, and smoking, but not for drug consumption with *p* = 0.044 ( Table 3 ). There was a correlation between the concentration of TNF-α and the incidence of periodontitis in patients with type 2 DM with and without periodontitis ( Table 4 ).

**Table 1:** Correlation between different characteristics and the incidence of periodontitis in type 2 diabetes mellitus patients with and without periodontitis

Diabetes mellitus						
Variables: characteristics	With periodontitis			Without periodontitis		*p* -Value
		*n*	%	*n*	%	
Length of suffering	>5 y	33	70.2	14	29.8	0.157
	≤5 y	24	55.8	19	44.2	
Family history	Positive	43	67.2	21	32.8	0.234
	Negative	14	53.8	12	46.2	
Ages	>61	29	54.7	24	45.3	0.042
	≤61	28	75.5	9	24.3	

**Table 2:** Correlation between behavioral (predisposing, enabling, and reinforcing) factors and the incidence of periodontitis in type 2 diabetes mellitus patients with and without periodontitis

Diabetes mellitus			
Variables: behavior	With periodontitis	Without periodontitis	*p* -Value
	Mean ± SD	Mean ± SD	
Predisposing factor	126.76 ± 17.066	129.76 ± 12.659	0.000
Enabling factor	16.05 ± 12.659	16.48 ± 2.431	0.445
Reinforcing factor	16.05 ± 2.808	22.12 ± 2.891	0.031

**Table 3:** Correlation between lifestyle and the incidence of periodontitis in type 2 diabetes mellitus patients with and without periodontitis

	Diabetes mellitus					
Variables: lifestyle	With periodontitis			Without periodontitis		*p* -Value
		*n*	%	*n*	%	
Food style	Poor	17	81.0	4	19.0	0.056
	Good	40	58.0	29	42.0	
Physical activities	Poor	55	64.0	31	36.0	0.571
	Good	2	50.0	2	50.0	
Drug consumption	Poor	26	76.5	8	23.5	0.044
	Good	31	55.4	25	44.6	
Management of oral cavity	Poor	23	65.7	12	34.3	0.708
	Good	34	61.8	21	38.2	
Smoking	Smoking	15	70.0	5	30.0	0.220
	Nonsmoking	42	61.4	28	38.8	

**Table 4:** Correlation between concentration of TNF-α and the incidence of periodontitis in type 2 DM patients with and without periodontitis

Diabetes mellitus					
Variable concentration of TNF-α levels	*N*	With periodontitis (mean ± SD)	*N*	Without periodontitis (mean ± SD)	*p* -Value
	57	0.628 ± 0.123	33	0.457 ± 0.097	0

Abbreviations: DM, diabetes mellitus; SD, standard deviation; TNF-α, tumor necrosis factor-α.

## Discussion

The occurrence of periodontitis in patients with type 2 DM increases with age due to the decrease in the physiological function of the body. According to Moore et al, the cumulative damage of a person's periodontal tissue gets worse with increasing age. Pancreatic β cells begin to shrink continuously which results in reduced insulin secretion and also reduced receptor sensitivity. 13 14 However, the long duration of diabetes and a family history of type 2 DM are factors that aggravate type 2 diabetes, but they are not significantly associated with the incidence of periodontitis in type 2 DM patients.

Type 2 DM is closely related to the behavior and lifestyle of the patients. One of the models commonly used to diagnose, treat, and prevent the disease is the PRECEDE–PROCEED model. 15 However, only predisposition and reinforcement were found to have a significant correlation in this study, while the supporting factors were not. The factors represented by insurance ownership and the respondent's financial ability were not always proportional to the respondent's ability in controlling type 2 DM. Predisposition and reinforcement factors were significantly correlated in this study, especially in the consumption of antidiabetes drugs. The reinforcing factor had a significant correlation with the incidence of periodontitis in patients with type 2 DM. The findings in this study indicated that significant family and health care staff support provided fundamental changes to the lifestyle of people with type 2 DM to be better so that the disease can be controlled.

Physical activity is a movement that is performed by the skeletal muscles that require energy. Exercise, however, is a planned, structured, repetitive, and intentional movement intended to improve or maintain physical fitness. Exercise improves blood glucose management in type 2 diabetes, reduces cardiovascular risk factors, contributes to weight loss, and improves well-being. 16 Regular exercise can save or delay the development of type 2 diabetes. 14 The demanding situation is related to blood glucose control range with diabetes type, hobby type, and the presence of diabetes-related complications. 12 17

Physical activity referred to in the study was the activity with a duration of at least 30 minutes four to seven times a week. The significant difference found in the results of this study was because the physical activity was dominated by the elderly patients performing physical activities for less than a minimum duration of 30 minutes four to seven times a week. Physical activity can increase the sensitivity of insulin receptors so that glucose can be converted into energy. When physical activity is performed, the muscles in the body will react by using stored glucose.

The level of periodontal damage in diabetics is influenced by glycemic control, that is the consumption of antidiabetes drugs and the individual's immune system. Poor glycemic control and increased formation of AGE products cause oxidative stress to the gingiva, which exacerbates periodontal tissue damage. Significant support from the family and health care staff was able to provide the best glycemic control with regular consumption of antidiabetic drugs, and reduced periodontal tissue damage.

The behavior of oral cavity care in type 2 DM patients was found not to have a significant correlation with the incidence of periodontitis. Two of five studies stated that there were significant changes in glucose levels in type 2 DM patients after periodontal treatment. This shows that oral cavity care in type 2 DM patients is not enough just by brushing the teeth, but with additional periodontal treatment in the form of scaling and root planning. 18

Smoking behavior in people with type 2 DM has a significant relationship with the incidence of periodontitis. Smoking is a risk factor for the development of periodontal disease and alveolar bone loss is 20 times higher in type 2 DM patients. 19 Type 2 DM patients who smoke have a deeper periodontal pocket than those who do not smoke. The nicotine in cigarettes has a short vasoconstrictive effect on the oral mucosa and cell metabolism. Smoking behavior was also found to support microbial growth in periodontal tissue. Smoking is also known to affect neutrophils and collagen, exacerbating periodontal conditions in people with type 2 DM.

Analysis of TNF-α levels in this study showed a significant correlation between TNF-α concentrations and the incidence of periodontitis in type 2 DM patients with and without periodontitis. The results of this study confirmed a previous study that found that TNF-α had a close relationship with periodontitis and type 2 DM. 20 TNF-α is produced as an inflammatory response in periodontitis and is an important additional factor in insulin sensitivity in type 2 DM. 21 22 The high levels of inflammatory mediators, such as TNF-α, contribute to increased insulin resistance. In addition, periodontal bacteria can migrate to the liver, inhibit insulin signaling, and result in decreased glycogen synthesis. 23 Recent systematic reviews have shown that periodontal therapy can positively influence DM control. 24 Age has a significant effect on TNF-α in patients with periodontitis and types 2 DM. 25

The results of research performed on rats showed that TNF-α levels decreased in periodontic experimental animals that received DM and dietary treatment. 26 In addition, based on the results of a study, 27 it was found that TNF-α had a significant relationship with periodontitis and type 2 DM in patients who received treatment. There was a decrease in TNF-α levels after drug consumption.

The limitation of this study was that the information obtained about the respondents, such as behavior and several other factors, was obtained by using a questionnaire, so that there was a possibility that the respondent did not answer according to reality. However, an anticipatory effort had been made to confirm the results by carrying out measurement using CPITN to assess the amount of bleeding on probing and the depth of the pocket. In a study conducted on rats, TNF-α levels decreased in those animals which experienced periodontitis and received DM and dietary treatment. It was found that TNF-α had a significant relationship in patients with periodontitis and type 2 diabetes who received treatment. It appeared that TNF-α levels in GCF and serum before treatment were higher in the GDM group than in the control group. Thus, TNF-α examination can improve understanding of the pathogenesis of periodontitis and GDM and its assessment in the treatment process can result in better disease control.

## Conclusion

High levels of the inflammatory mediator, such as TNF-α, might contribute to an increase in insulin resistance. Inflammation will be more severe in the presence of hyperglycemia because hyperglycemia causes an increase in proinflammatory cytokines like TNF-α. TNF-α stimulates osteoclasts to mature and actively absorb bone. Periodontal bacteria can translocate to the liver, inhibit insulin signaling, and result in decreased glycogen synthesis. Poor glycemic control will increase the formation of AGE products that can induce oxidative stress on the gingiva, thereby aggravating damage to periodontal tissue. In patients with type 2 DM, periodontitis is prevented not only by regularly maintaining oral cavity health but also by controlling blood sugar levels by regularly consuming the drugs. Knowledge is a factor that affects adherence of people with type 2 DM in consuming drugs regularly, strengthened by family support which will ultimately play a role in reducing TNF-α levels.
